# Distribution of endothelial progenitor cells in tissues from patients with gastric cancer

**DOI:** 10.3892/ol.2014.1944

**Published:** 2014-03-06

**Authors:** XIAO-QIN HA, MAN ZHAO, XIAO-YUN LI, JUN-HUA PENG, JU-ZI DONG, ZHI-YUN DENG, HONG-BIN ZHAO, YONG ZHAO, YUAN-YUAN ZHANG

**Affiliations:** Department of Clinical Laboratory, Lanzhou Military Command General Hospital of the People’s Liberation Army; Key Laboratory of Stem Cell and Gene Medicine of Gansu Province, Lanzhou, Gansu 730050, P.R. China

**Keywords:** endothelial progenitor cells, gastric cancer, distribution

## Abstract

It is accepted that endothelial progenitor cells (EPCs) are recruited into tumor sites and take part in the neovascularization of tumors. However, few articles have discussed the specific distribution of EPCs *in vivo* in tissues of gastric cancer patients. For this reason, the present study sought to elucidate EPC distribution *in vivo* in tissues of patients with gastric cancer. Fresh tumor tissues were collected from 26 newly diagnosed patients with histologically confirmed gastric cancer (mean age, 51 years; range, 21–81 years; 7 females, 19 males). All patients were treated surgically with curative intent. One portion of the fresh tissues was prepared for flow cytometric analysis and another was immediately snap frozen in liquid nitrogen and stored at −80°C for later use in quantitative polymerase chain reaction. The analysis was based on two groups of tissues, namely the cancer group and cancer-adjacent group. The presence of CD34/CD133 double-positive cells was determined in cancer-adjacent and cancer tissues by flow cytometry. The analysis revealed that the total number of EPCs in cancer tissue was slightly greater than the number in the cancer-adjacent tissue, but not to the point of statistical significance. The number of EPCs in cancer-adjacent and cancer tissues of patients with early-stage gastric cancer was higher than the EPC count in late-stage gastric cancer patients, and significant differences were identified in the number of EPCs in cancer tissue between patients of different tumor stages. Levels of cluster of differentiation (CD)34, CD133 and vascular endothelial growth factor receptor 2 were not significantly different in cancer-adjacent tissue compared with cancer tissue. These results suggest that cancer-adjacent and cancer tissue of gastric cancer patients may be used as a reference index in the clinical and pathological staging of tumors.

## Introduction

It is well known that the growth of new blood vessels is a component of certain pathological conditions, including tumor growth and metastasis. Previous experimental studies have suggested that bone marrow-derived circulating endothelial progenitor cells (EPCs) migrate to neovascularization sites and differentiate into endothelial cells *in situ*, a process termed vasculogenesis (1,2). Whether bone marrow-derived EPCs participate in the progression of gastric cancer has not yet been evaluated.

Bone marrow-derived EPCs were first isolated from whole blood using magnetic microbeads coated with cluster of differentiation (CD)34 antibody by Asahara *et al* in 1997 (2). EPCs are a group of immature endothelial cells with proliferation and differentiation potential, and are derived from hematopoietic stem/progenitor cells, which are also the precursor of hematopoietic cells. It is widely accepted that CD34^+^, CD133^+^ and vascular endothelial growth factor receptor 2^+^ (VEGFR-2, also known as kinase insert domain receptor or Flk1) cells are EPCs (3). EPCs are important initiators of vasculogenesis in the process of tumor neovascularization. Increased levels of EPCs in peripheral blood were identified in patients with pancreatic carcinoma (4), malignant gliomas (5), and ovarian (6), non-small cell lung (7) and gastric (8) cancer. Consequently, the level of EPCs has been proposed as a novel biomarker for the diagnosis and monitoring of these lesions. Although these studies have prompted trials to use EPCs in this way, the specific distribution of EPCs *in vivo*, and whether the number of EPCs is associated with tumor stage in cancer tissue, has seldom been discussed. The present study investigated the distribution of EPCs *in vivo*, providing valuable information for clinical diagnosis, detection and treatment of cancer.

## Materials and methods

### Patients

Patients were recruited from the Lanzhou Military Command General Hospital of the People’s Liberation Army (Lanzhou, China). The ethics committee of Lanzhou Military Command General Hospital of the People’s Liberation Army (Lanzhou, China) approved the study, and written informed consent was obtained from all study participants. Fresh tumor tissues from 26 newly diagnosed patients with histologically confirmed gastric cancer were collected. All patients were treated surgically with curative intent. The patients had no additional malignant, inflammatory or ischemic disease, or wounds or ulcers that could influence the number of EPCs. One portion of the fresh tissues was prepared for flow cytometric analysis and another was immediately snap frozen in liquid nitrogen and stored at −80°C for later use in the study. The entire group of patients included 19 male cases and 7 female cases aged 21–81 years (mean, 51 years; median, 55 years; ≥55 years of age in 13 cases). No patient had received radiotherapy or chemotherapy prior to tumor excision. In addition, normal gastric cancer tissue 5 cm from the tumor margin was obtained from each patient for comparison.

### Sample preparation

Fresh tissue (50 mg) was washed with saline (10% heparin), then soaked in saline for ~40 min. With ophthalmic scissors the tissue was cut into small pieces ~1 mm^3^ and digested with 1 ml trypsin at 37°C for 15 min, gentle agitation every 3 min during the process. Digestion was terminated with 2 ml 10% fetal calf serum (Clonetics, Cambrex, MD, USA). Large clumps of tissue and connective tissue were removed using a 200-mesh filter and the cell suspension was collected. The solution was centrifuged at low speed (1,300 × g) for 10 min, the cells were collected and the supernatant was discarded. The precipitate was washed with 1 ml phosphate-buffered saline (PBS) and resuspended in 200 *μ*l PBS buffer containing CD34 (BD Biosciences, San Diego, CA, USA), CD133 (Miltenyi Biotec, Bergisch Gladbach, Germany), VEGFR-2 (R&D Systems Inc., Minneapolis, MN, USA) and 5 *μ*l monoclonal fluorescent antibody. The mixture was incubated for 30 min according to the manufacturer’s recommendations (BD Biosciences). Samples were fixed in 1% formaldehyde and analyzed using a FACSCalibur flow cytometer (BD Biosciences). The control and experimental groups used the same processing methods.

### Flow cytometric analysis

EPCs were identified by the expression of CD34, VEGFR-2 and CD133. A volume of 200 *μ*l single cell suspension was incubated for 30 min in the dark with fluorescein isothiocynate-labeled monoclonal antibodies from mouse ascites against human CD34, allophycocyanin-labeled monoclonal antibodies from mouse ascites against human CD133 and phycoerythrin-labeled monoclonal antibodies from mouse ascites against human VEGFR-2. Mouse isotype-identical antibodies served as controls (BD Biosciences). For analysis, 200,000 cells within the leukocyte gate were acquired using a FACSCalibur analyzer and data were processed using FACSDiva software (both purchased from BD Biosciences). The percentage of cancer-adjacent and cancer tissue EPCs was determined using the three-color antibody panel previously described and an appropriate gating strategy. CD45-dim cells positive for VEGFR-2 with low to medium forward- and side-scattered light and positive for CD34 and CD133 were considered EPCs. The absolute number of cells (cells/*μ*l) was calculated with the following formula: Percentage of cells × total nucleated cells/100 (10). As a similar pattern of modifications following gastric cancer surgery for all three types of EPC (CD34^+^/ VEGFR-2^+^, CD133^+^/ VEGFR-2^+^ and CD34^+^/CD133^+^/ VEGFR-2^+^) was observed, only data on EPCs characterized as CD34^+^/CD133^+^ has been included.

### Quantitative polymerase chain reaction (qPCR)

Total RNA was extracted from tissue that had been frozen in liquid nitrogen immediately following surgery, using TRIzol (Invitrogen Life Technologies, Carlsbad, CA, USA), and cDNA was synthesized from each tissue sample with M-MLV reverse transcriptase (Invitrogen Life Technologies) according to the manufacturer’s instructions. qPCR (20 *μ*l reactions) with SYBR GreenER qPCR SuperMix Universal (Invitrogen Life Technologies) was performed in triplicate using a 7300 Fast Real Time PCR system (Stratagene, La Jolla, CA, USA). A no-template reaction (RNA replaced with water) was used as a negative control. Target gene expression was determined using the 2^−ΔΔCt^ method and normalized using β-actin as an internal control. To determine PCR amplification efficiency, standard curves were constructed using different concentrations of template cDNA for VEGFR-2, CD34, CD133 and β-actin. For all genes, the correlation coefficient of the standard curve was ≥0.96, and the amplification efficiency was almost 1.0. The primer sequences used for qPCR are listed in Table I.

### Statistical analysis

Results are presented as mean values ± standard deviation. Statistical analyses were performed using SPSS software (version 17.0; SPSS Japan Inc., Tokyo, Japan). Differences between groups were calculated using the Mann-Whitney U test and two-way analysis of variance, and these were later evaluated by post hoc analysis. P<0.05 was considered to indicate a statistically significant difference.

## Results

### EPCs and clinical data

**Figure 1:**
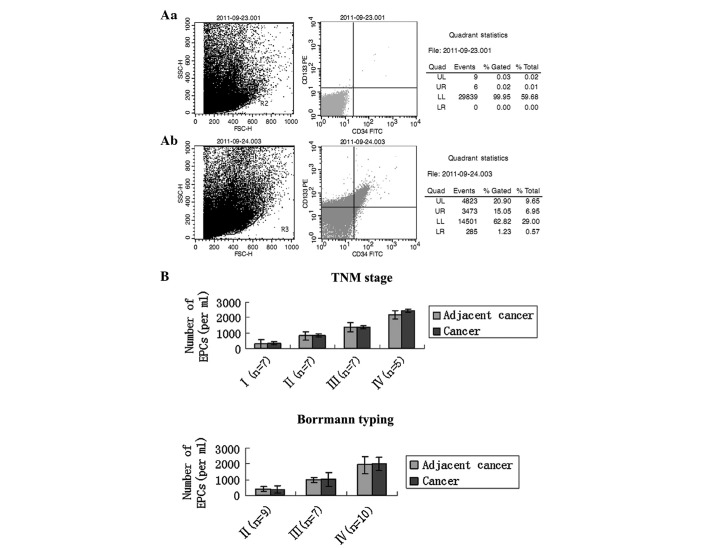
(A) Representative flow cytometric analysis from a patient with gastric cancer. Left, flow cytometry gating; (Aa) isotype-negative control for flow cytometry; (Ab) representative flow cytometric analysis for determining the number of CD34/CD133 double-positive cells. (B) Comparison of circulating EPC levels in cancer-adjacent tissue and cancer tissue in different TNM stages and Borrmann types of gastric cancer patients. Data are expressed as the mean ± standard deviation. EPCs, endothelial progenitor cells; CD, cluster of differentiation.

Although no clear definition of EPC exists, based on previous studies using flow cytometry, this study determined the numbers of CD34/CD133 double-positive cells in cancer-adjacent and cancer tissue of gastric cancer patients (Fig. 1A). Additionally, the number of VEGFR-2^+^/CD133^+^ cells was measured, corresponding to a subfraction of immature EPCs. However, as VEGFR-2^+^/CD133^+^ and CD34^+^ EPC counts did not differ from each other significantly (P>0.1 for all analyses; data not shown), in further experiments, only the levels of EPCs with the latter phenotype were evaluated, in accordance with previous studies (11).

For the 26 patients, the results revealed that the mean number of EPCs in cancer tissue was marginally greater than the number in the cancer-adjacent tissue, but with no statistical significance in the age and gender groups (Table II). The number of EPCs in cancer-adjacent tissue of patients with early-stage gastric cancer was lower than the number in patients with late-stage gastric cancer (TNM stage I/II, n=14, 580±292.3 cells/mm^2^ vs. stage III/IV, n=12, 1,816±590.3 cells/mm^2^), and significant differences were identified in the numbers of EPCs in cancer tissue at different tumor stages (TNM stage I, n=7, 340±105.8 cells/mm^2^; stage II, n=7, 821±197.3 cells/mm^2^; stage III, n=7, 1,360±196.6 cells/mm^2^; stage IV, n=5, 2,455±163.5 cells/mm^2^). However, the number of EPCs in cancer-adjacent tissue at each TNM stage was no higher than that in cancer tissue. Further analysis revealed that Borrmann stage and histological type were also associated with the number of EPCs (P<0.05) (Fig. 1B).

### EPC markers in cancer-adjacent and cancer tissue determined by qPCR

**Figure 2:**
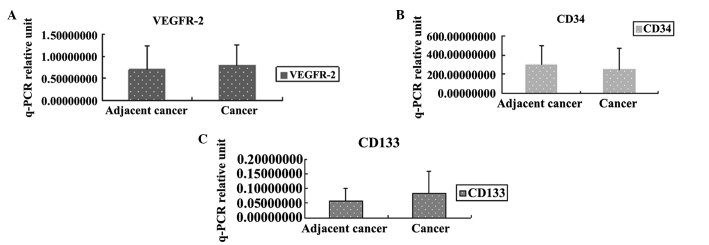
Relative gene expression levels of (A) VEGFR2, (B) CD34 and (C) CD133 in cancer-adjacent and cancer tissue of gastric cancer patients as determined by qPCR. VEGFR-2, vascular endothelial growth factor receptor 2; CD, cluster of differentiation; qPCR, quantitative polymerase chain reaction.

Cancer tissue CD34, CD133 and VEGFR-2 mRNA levels were determined by qPCR. Levels of CD34, CD133, VEGFR-2 were not significantly different in cancer-adjacent tissue compared with cancer tissue in the gastric cancer patients (Fig. 2).

## Discussion

Gastric cancer is the fourth most common type of cancer worldwide (12). According to Parkin *et al* (13), gastric cancer has the second and fourth highest mortality rate for men and women, respectively (13). The prognosis of gastric cancer patients is poor, with a five-year survival rate of ~20% (12,14). Surgical resection with curative aim is the principal treatment for gastric cancer, and the suitability of surgical resection is decided based on the tumor stage of the patient (15).

At present, the role of EPCs in tumors is a major focus in the field of oncology. However, few articles have discussed the specific distribution of EPCs *in vivo*. For this reason, the present study sought to elucidate EPC distribution *in vivo* by assessing the number of EPCs in cancer tissue excised from gastric cancer patients. In addition, the study analyzed the association between EPCs and tumor stage in an attempt to identify more reliable diagnostic methods for tumors.

Since EPCs were first reported (2), it has been recognised that EPCs correlate closely with neovascular formation. Preliminary reports have demonstrated that circulating EPCs may be incorporated into tumor vascularization and may correlate with neovascularization (1). The existence of a BM reservoir and its contribution to neovascular formation are of great interest (16) and may be used as an index in order to detect cancer progression (15). However, it remains uncertain as to whether EPCs are present in patients with cancer and what roles they may play.

EPCs are derived from BM-derived hematopoietic cells, which may be induced into forming ECs which in turn contribute to neovessel formation (18). Tumor cytokines, involved in the formation of CEPs, are derived from EPCs in the peripheral blood circulation. Subsequently the EPCs gradually infiltrate the tumor vascular bed and are incorporated into neovessels (16).

In the present study, EPCs were measured by fluorescence-activated cell analysis of fresh cancer and cancer-adjacent tissue and defined by the expression of surface markers CD34^+^/VEGFR-2^+^ and CD133^+^/VEGFR-2^+^ (19). Experiments on the migration of EPCs toward the site of neovascularization were carried out in order to examine the significance of EPCs in cancer tissue. The present study provides evidence that BM-derived EPCs, defined by the cell surface expression of CD34 and CD133, differentiate into mature endothelial cells and contribute structurally and functionally to tumor neovascularization.

The purpose of the study was to observe whether the number of EPCs in gastric cancer and paracancerous tissue differed. The results revealed no significant differences between gastric cancer tissue and paracancerous tissue. According to previous reports, hypoxia in the tumor tissue micro-environment is the initiating factor for EPCs to participate in tumor growth. Tumors may produce high levels of hypoxia-inducible factor (HIF) 1α, which induces the production of VEGF and stromal derived factor 1α. VEGF is one of the most important target genes of HIF-1α and it is also a main factor in the creation of new blood vessels in tumors. Tumor cells, alongside immune cells and tumor fibroblasts, can secrete VEGF directly. VEGF mobilizes VEGFR-2-positive EPCs to the peripheral blood circulation, which then migrate to the tumor site to assist in the formation of new blood vessels (20). This may suggest that changes in the number of EPCs in gastric cancer tissue and paracancerous tissue may not be significantly different.

The stage of the tumor is extremely important for treatment and prognosis, and this study demonstrated incidentally that EPC levels correlate with tumor clinical and pathological staging. The number of EPCs is significantly correlated with tumor TNM stage, Borrmann stage and degree of differentiation. Thus, testing the number of EPCs in gastric cancer tissue and paracancerous tissue may provide indicators for the clinical and pathological diagnosis of gastric cancer.

To date no unique marker for EPCs has been reported. Additionally there is no consensus on the definition of EPCs. Therefore, building a functionally characterized dataset rare putative EPCs based on FACs phenotypes is difficult, making comparisons with other published work difficult as there is no statndard. Therefore, it is necessary to locate an effective method for the enumeration of circulating EPCs (21,22). With a better understanding of EPCs, we can approach the role of EPCs in tumor progression. The present study demonstrates that EPC levels are significantly increased and are correlated with cancer stage in the cancer tissue and paracancerous tissue of gastric cancer patients. Furthermore, although our data suggest the participation of EPCs in tumor growth in gastric cancer, it is not clear whether these cells are essential for this process. Further investigation is warranted for the potential application of EPCs in monitoring disease progression or as targets for gastric cancer treatment.

These results suggest that EPC count in cancer-adjacent and cancer tissue of gastric cancer patients can be used as a reference index in the clinical and pathological staging of tumors. Additional prospective investigations in a large population are required to confirm these findings.

## Figures and Tables

**Table I:** Primer sequences used for qPCR.

Primer	Sense, 5′-3′	Antisense, 5′-3′
CD34	CTCTCACCTGTACTCTTCC	CAGCTGGTGATAAGGGTTA
CD133 (9)	TGGATGCAGAACTTGACAACGT	ATACCTGCTACGACAGTCGTGGT
VEGFR-2	CACCACTCA AACGCTGACATGTA	GCTCGTTGGCGCACTCTT
β-actin	TCTGGCACCACACCTTCTAC	CTCCTTAATGTCACGCACGATTTC

CD, cluster of differentiation; VEGFR-2, vascular endothelial growth factor receptor 2.

**Table II:** Baseline characteristics and perioperative data of patients.

		Endothelial progenitor cells, n		
			
Data	Patients, n	Cancer-adjacent	Cancer	P-value
Age, years				>0.05
<55	13	984±618.4	988±622.5	
≥55	13	1318±889.7	1189±833.5	
Gender				>0.05
Male	7	1102±777.8	1042±715.5	
Female	19	1169±787.2	1106±751.2	
TNM stage				<0.001
I	7	299±98.2	340±105.8	
II	7	817±206.4	821±197.3	
III	7	1364±367.8	1360±196.6	
IV	5	2187±415.7	2455±163.5	
Borrmann stage				<0.001
II	9	398±150.9	378±225.7	
III	7	976±166.7	1018±442.9	
IV	10	1951±552.2	2010±427.4	
Differentiation status				<0.001
Well differentiated	7	364±119.3	319±106.3	
Moderately differentiated	7	1047±291.7	986±258.8	
Poorly differentiated	12	2068±556.3	1986±429.8	

Cells numbers are expressed as mean ± standard deviation.
